# Aplastic anemia secondary to adjuvant Osimertinib therapy: a case report and a review of literature

**DOI:** 10.3389/fonc.2024.1275275

**Published:** 2024-02-22

**Authors:** Ahmed Abdalhadi, Nabil E. Omar, Samah Kohla, Hassan Aakel, Yeslem Ekeibed, Reyad Mohsen

**Affiliations:** ^1^ Medical Oncology, National Center for Cancer Care and Research (NCCCR), Hamad Medical Corporation, Doha, Qatar; ^2^ Pharmacy Department, National Center for Cancer Care and Research (NCCCR), Hamad Medical Corporation, Doha, Qatar; ^3^ Health Sciences Program, Clinical and Population Health Research, College of Pharmacy, Qatar University, Doha, Qatar; ^4^ Lab Medicine and Pathology, Hematopathology, National Center for Cancer Care and Research (NCCCR), Hamad Medical Corporation, Doha, Qatar; ^5^ Medicine, Weill Cornell Medicine, Doha, Qatar; ^6^ Clinical Hematology, National Center for Cancer Care and Research (NCCCR), Hamad Medical Corporation, Doha, Qatar

**Keywords:** EGFR, TKI, SCLC, AA, lung adecarcinoma

## Abstract

Aplastic anemia is a rare hematological disorder characterized by suppressed hematopoiesis and pancytopenia. Although several drugs have been associated with aplastic anemia, its occurrence in response to Osimertinib, a third-generation epidermal growth factor receptor (EGFR) tyrosine kinase inhibitor (TKI), is extremely rare. We present a case report of a 63-year-old patient with locally advanced non-small cell lung cancer (NSCLC) who developed aplastic anemia following adjuvant treatment with Osimertinib. Extensive investigations ruled out infectious etiology, and the absence of bone marrow involvement or other identifiable causes suggested a drug-induced etiology, specifically Osimertinib. This case report emphasizes the importance of recognizing this adverse event and considering it as a potential complication of Osimertinib therapy. Vigilant monitoring and prompt management are essential for optimizing patient outcomes. Further studies are needed to better understand the risk factors, underlying mechanisms, and management strategies for Osimertinib-induced aplastic anemia in the adjuvant settings.

## Introduction

Lung cancer ranked the second most common cancer worldwide (1). It affects both men and women, with men experiencing it more frequently than women. Lung cancer is the leading cause of cancer-related deaths globally for both genders (2).

There are two main types of lung cancer: small cell lung cancer (SCLC) and non-small cell lung cancer (NSCLC). NSCLC is more common, accounting for approximately 85% of cases. The most prevalent subtype of NSCLC is adenocarcinoma, followed by squamous cell carcinoma and large cell carcinoma (3, 4).

The management of lung cancer depends on various factors, including the histopathologic subtype as a pivotal factor, stage of the disease, performance status of the patient, and the presence of specific genetic mutations. Treatment options encompass surgery, radiation therapy, chemotherapy, targeted therapy, and immunotherapy (5).

Epidermal growth factor receptor (EGFR) mutations are frequently found in NSCLC, especially in patients who are non-smokers or have a history of light smoking. They occur in 49.1% of Asian patients or 12.8% of European patients (6). Certain drugs known as EGFR inhibitors, such as erlotinib, gefitinib, afatinib, and Osimertinib, are used to block the activity of the mutated EGFR protein. This inhibition leads to reduced cancer cell growth and improved patient outcomes (7).

Osimertinib is a third-generation EGFR tyrosine kinase inhibitor (TKI) that selectively targets tumors with EGFR mutations, including exon 19 deletions and exon 21 L858R mutations. Exon 19 deletions and the L858R point mutation are the most common mutations and identified as reliable indicators for a positive clinical response to EGFR TKI (8). It is approved as a first-line treatment for EGFR mutant metastatic NSCLC. Osimertinib is approved for the treatment of locally advanced or metastatic NSCLC that has developed resistance to other EGFR TKIs due to the acquisition of the T790M mutation (9).

In 2020, the ADAURA trial demonstrated that Osimertinib significantly prolongs disease-free survival compared to placebo in stage IB to IIIA EGFR mutation-positive NSCLC. It showed a significant reduction in the risk of disease recurrence or death (10). Moreover, according to the latest findings from the BLOOM study and other sporadic case reports, Osimertinib has shown efficacy in treating leptomeningeal carcinomatosis, irrespective of the presence of T790M mutation (11–13).

Aplastic anemia (AA) is a bone marrow disorder characterized by a failure in the bone marrow production of red blood cells, white blood cells, and platelets. It is a rare disorder with an incidence of around 0.6 to 6.1 cases per million people per year (14, 15). It is a life-threatening disorder, if untreated, with current 5-year overall survival 70 to 80% compared to 10 to 20% in the 1960s (16).

Bone marrow failure can occur through three primary mechanisms: direct harm to the marrow, constitutional syndrome, and immune AA. Direct damage to the bone marrow is typically iatrogenic, resulting from various causes such as medications, chemotherapy, and radiation therapy (17). The exact cause of AA remains unknown, and various hypotheses propose genetic and environmental factors as potential triggers (18). An immune mechanism is primarily associated with almost all sporadic cases of AA, particularly those that are severe and acute (17).

In the context of TKI therapy, AA can be considered an adverse event of the treatment (19).

AA associated with Osimertinib has been previously observed in patients with metastatic disease (20–23). To our knowledge, there are no published cases reporting AA secondary to Osimertinib specifically as adjuvant treatment.

## Case presentation

A 63-year-old Filipino ex-smoker female patient with a history of 20 pack-years presented to a pulmonology clinic due to an abnormal chest x-ray. She does not have any past medical history of chronic illnesses, and she does not use any medications. Further imaging with a chest CT scan revealed a plural-based nodule in the left lower lung lobe, a small nodule in the right lower gland lobe, and hypodense lesions in the right liver lobe. A biopsy confirmed the diagnosis of pulmonary adenocarcinoma, acinar variant, with an EGFR exon 19 deletion. The initial staging was cT4N0M0 (Stage IIIA) and the patient underwent video-assisted thoracoscopic surgery (VATS) but had to abort the procedure due to a positive biopsy of the pleural nodule. The staging was subsequently revised to cT4N0M1a (stage IVA) but the patient was still a surgical candidate.

**Figure 1:**
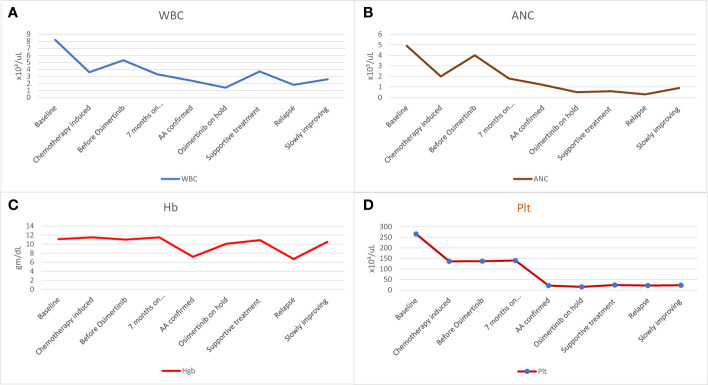
The patient’s hematologic findings. **(A)** WBC count before starting, during, and after stopping Osimertinib, **(B)** ANC before starting, during, and after stopping Osimertinib, **(C)** Hb level before starting, during, and after stopping Osimertinib, and **(D)** Platelet counts before starting, during and after stopping Osimertinib. AA, aplastic anemia; ANC, absolute neutrophil count; Hb, hemoglobin; Plt, platelets; WBC, white blood cell.

At initial presentation, the baseline complete blood count (CBC) showed white blood cell count (WBC) of 4.8 x10³/uL (reference range 4.0-10.0), absolute neutrophil count (ANC) of 2.5 x10³/uL (reference range 2.0 - 7.0), Hemoglobin (Hb) of 12.9 gm/dL (reference range 13.0-17.0) and platelets (PLTs) of 171 x10³/uL (reference range 150-400) (**Figure 1**).

The patient received four cycles of neoadjuvant chemotherapy (carboplatin AUC 5 plus pemetrexed 500 mg/m^2^), during which she experienced moderate neutropenia with ANC of 1.0 x10³/uL after the third cycle. Treatment with subcutaneous filgrastim 300 mcg for three days improved neutropenia to the normal level with ANC of 2.3 x10³/uL (**Figure 1B**).

Following neoadjuvant chemotherapy, the patient underwent mediastinoscopic lymph node biopsy, VATS, and left lower lobectomy. The final pathological staging was ypT2a, N2. Considering the stage and the presence of EGFR exon 19 deletion, the patient was scheduled to receive adjuvant treatment with Osimertinib 80 mg daily.

Before initiating Osimertinib, the patient’s blood tests were within normal range (**Figure 1**). However, one week after starting Osimertinib, mild pancytopenia was observed, with a decrease in WBC to 3.3 x10³/uL, ANC to 1.8 x10³/uL, Hb to 11.5 gm/dL and PLTs to 140 x10³/uL (**Figure 1**). Despite these findings, Osimertinib was continued. She was doing well but was not attending her regular follow-up appointments and no further blood tests were conducted thereafter.

**Figure 2:**
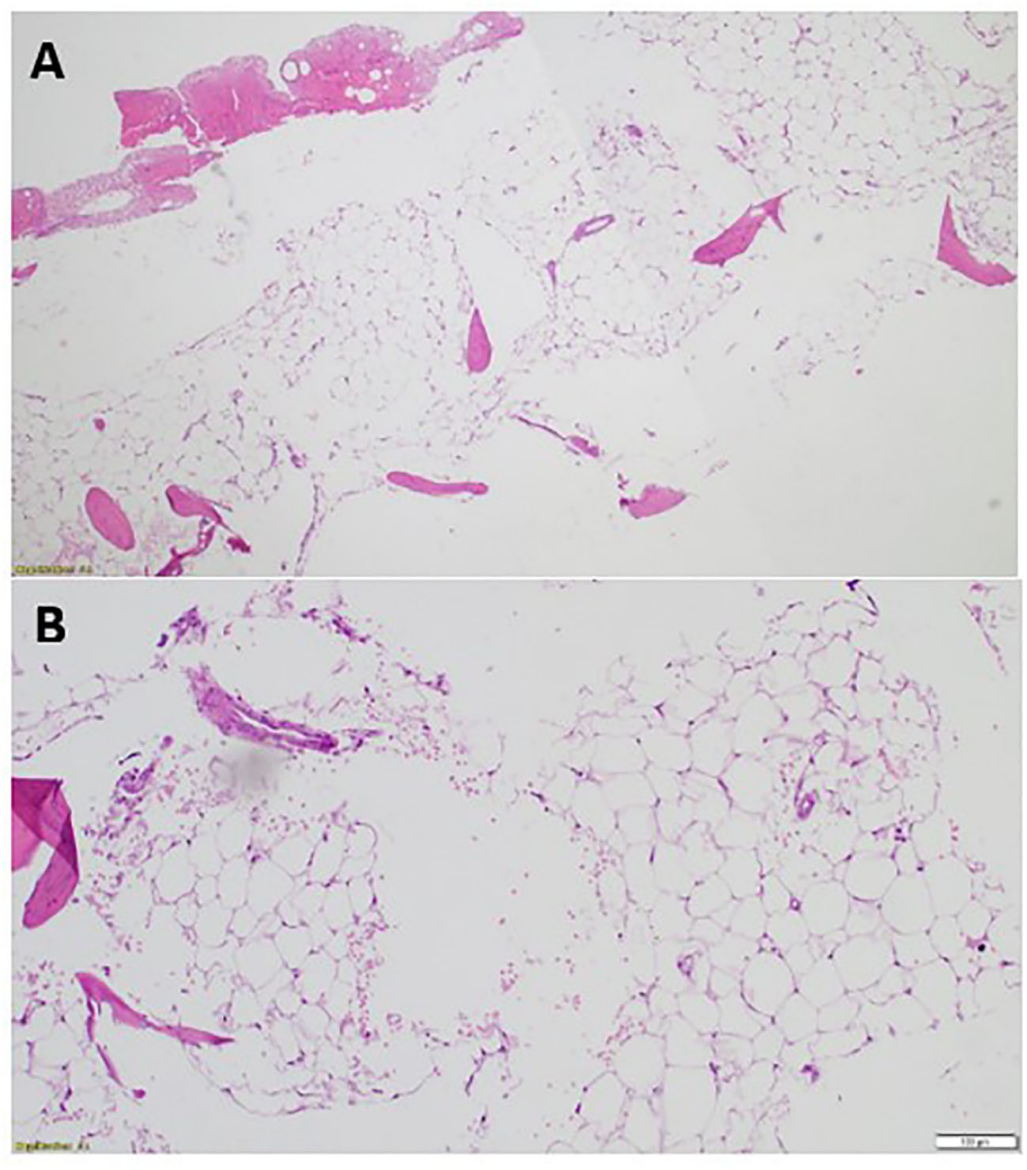
The patient’s bone marrow biopsy findings. **(A)** Bone marrow trephine biopsy (low power) is markedly hypocellular with <5% cellularity showing marked depression of the trilineage hematopoiesis [Hematoxylin and eosin, H&E, staining x4]. **(B)** Bone marrow trephine biopsy (high power) showing fatty replaced intertrabecular spaces with few nucleated cells and almost absent megakaryocytes [H&E staining x20].

After seven months on Osimertinib treatment, the blood investigations showed that the patient developed progressive pancytopenia characterized by moderate anemia with Hb of 9.3 gm/dL, reticulocytopenia with absolute reticulocyte count of 23 x10³/uL (reference range 50-100), leukopenia with severe neutropenia (WBC of 1.4 x10³/uL and ANC of 0.5 x10³/uL) and severe thrombocytopenia (PLTs of 12 x10³/uL). She was admitted to the hospital with a fever and a drop of Hb down to 7.2 gm/dL, necessitating a blood transfusion. No respiratory viral or bacterial infection was identified, and the patient’s condition improved spontaneously. However, her blood parameters did not fully recover, prompting further investigation with a bone marrow aspiration and biopsy. Bone marrow aspirate was significantly hemodiluted and not contributory. Bone marrow biopsy was markedly hypocellular (< 5% cellularity) with markedly suppressed trilineage hemopoiesis and almost absent megakaryocytes. No abnormal infiltrates, lymphoid aggregate, or granuloma was identified (**Figure 2**). No increase in CD34-positive cells by immunohistochemistry and CK AE1/AE3 was negative.

The overall peripheral blood and bone marrow biopsy findings confirmed the diagnosis of AA, most likely acquired AA.

A follow-up PET CT was done and showed no disease recurrence or bone metastasis. With the fact no active disease and the insight of current insult likely secondary to Osimertinib, it was decided to discontinue the remaining duration (3 years) of adjuvant treatment with Osimertinib.

Following discontinuation of Osimertinib, the patient’s blood parameters showed mild improvement one month later, with WBC of 1.9 x10³/uL, ANC of 0.7 x10³/uL, Hb of 9.3 gm/dL and PLTs of 24 x10³/uL (**Figure 1**). However, the levels remained below the normal range.

Subsequently, the patient was managed supportively through the administration of granulocyte-colony stimulating factor (G-CSF), along with frequent transfusions of platelets and red blood cells. Eltrombopag was introduced at a later stage, and no immunosuppressive medications have been given.

## Discussion

Osimertinib, an orally administered EGFR inhibitor, has received FDA approval for the treatment of NSCLC in both palliative and adjuvant settings (10, 24). It showed a significant improvement in progression and overall survival in metastatic disease, and brain metastasis. Additionally, in patients with stage IB to IIIA EGFR mutation–positive NSCLC who underwent adjuvant Osimertinib therapy, a substantial extension in disease-free survival was observed (10). Osimertinib is generally well-tolerated, but it can still have side effects. In comparison to first-generation TKIs like Erlotinib and Gefitinib, as well as second-generation TKIs like Afatinib; Osimertinib was associated with a lower frequency of side effects (25). However, when compared to other TKIs, Osimertinib does carry a higher risk of leukopenia and neutropenia. It is important to note that severe or life-threatening hematological toxicity with Osimertinib is rare (26).

The ADAURA trial is a double-blind, phase 3 study that involves patients with fully resected NSCLC carrying EGFR mutations. Participants were randomized in a 1:1 ratio to receive either Osimertinib at a daily dose of 80 mg or a placebo for a duration of 3 years (10). The safety analysis of this study included 337 patients in the Osimertinib group in the ADAURA trial, with adverse events reported in 329 patients (98%). Commonly reported adverse events were diarrhea, nausea, vomiting, rash, dry skin, fatigue, cough, and muscle and joint pain. Serious adverse events were reported in 54 patients in the Osimertinib group (10). However, it is important to note that hematological side effects, including AA, were not reported in the Osimertinib group. On the other hand, hematological side effects have been reported in patients with metastatic disease who received Osimertinib, with pancytopenia occurring in 22 patients based on the AURA3 trial and anemia developing in 34 patients out of 279 in the FLAURA trial (24, 27). Various factors can contribute to hematological disorders in advanced metastatic cases, including bone marrow infiltration, chemotherapy, radiotherapy, or immunotherapy (28–30).

This case highlights AA as a rare, yet serious adverse event of Osimertinib.

AA is characterized by a decrease in blood cell production in the bone marrow, leading to low levels of red blood cells, white blood cells, and platelets (31).

Certain drugs have been associated with the development of AA, including some antibiotics, nonsteroidal anti-inflammatory drugs, anticonvulsants, antithyroid drugs, and certain chemotherapy agents (31). However, drug-induced AA is relatively rare. The exact mechanisms by which drugs induce AA are not fully understood, but hypotheses such as immune-mediated, direct toxicity, and metabolic factors have been proposed (32).

There are two types of drug-induced bone marrow toxicity: dose-related reversible marrow aplasia and dose-independent idiosyncratic aplasia, which carries a higher mortality rate (17).

The exact mechanism by which Osimertinib may induce AA is unclear.

In the case presented, the patient experienced pancytopenia and severe neutropenia, putting her at an increased risk of infections. The absence of a respiratory viral or bacterial infection suggests that Osimertinib might have directly contributed to her AA rather than infectious causes. Information regarding this specific association is limited.

Our literature review identified four published case reports documenting the occurrence of AA associated with Osimertinib. Notably, these cases involved patients with advanced metastatic cancer. **Table 1** provides a summary of the key characteristics of these cases.

**Table 1:** A list of case reports of documented cases of Osimertinib-induced aplastic anemia in patients with lung cancer.

Ref. Number and Year of publication	Age at diagnosis, years	Gender	Previous Chemotherapy	Previous TKI	Medical co-morbidities	Dose	Interval between starting Osimertinib and diagnosis of aplastic anemia	Bone marrow biopsy performed	Treatment of AA	Osimertinib resumed	Death
Ref (20) ^2017^	78	F	Y	Y (gefitini)	No	80 mg	31 weeks	Y	Cyclosporine A 250 mg daily for 1 month	No	No
Ref (21) ^2018^	67	M	Y	Y (gefitini)	No	80 mg	19 weeks	Y	G-CSF, Eltrombopag	No	No
Ref (22) ^2020^	74	M	No	No	No	80 mg	21 weeks	Y	Steroid for 7 days with Filgrastim for 3 days	No	Y*
Ref (23) ^2022^	69	M	No	No	No	80 mg	10 days	Y	Filgrastim 480 mcg for 5 days	Y (after 2 months)	No

AA, Aplastic Anemia; TKI, Tyrosine Kinase Inhibitor; G-CSF, Granulocyte colony stimulating factor; M, Male; F, Female; Y, yes. * Cause of death not specified.

Among the reported cases, only one patient had Osimertinib resumed at a reduced dose of 40 mg. This patient demonstrated a positive response, with stable blood cell counts. In three cases, the development of pancytopenia occurred approximately 20 weeks after initiating Osimertinib treatment. This suggests potential dose-related toxicity, indicating the need for careful monitoring and dose adjustment in patients receiving Osimertinib to minimize the risk of hematological complications. Moreover, it has been observed that the risk of AA and other hematological side effects in patients who received Osimertinib may be higher in patients who have certain risk factors. These factors can include the presence of bone metastasis (the spread of cancer to the bone), previous exposure to chemotherapy, the presence of other coexisting medical conditions, and the use of Osimertinib for the treatment of metastatic advanced disease.

The management of AA includes various approaches. Supportive care measures, such as blood transfusions to address anemia and strategies to prevent infections, are employed. Non-transplant therapies, such as immunosuppression using anti-thymocyte globulin and cyclosporine A, are utilized. In certain cases, hematopoietic stem cell transplantation may be considered as a potentially curative option (33). It is worth mentioning that none of the published cases showed a response to treatment with steroids, Eltrombopag or immune suppressive medications, indicating the limited efficacy of these approaches in managing Osimertinib-induced AA.

Considering the patient’s persistent abnormal blood parameters after discontinuing Osimertinib, close monitoring of her condition is crucial, and further treatment options, including potential therapies for AA, should be discussed with a hematologist.

It is important to note that the development of aplastic anemia in response to Osimertinib is rare, and the benefits of Osimertinib in treating the patient’s lung cancer should be weighed against the potential risks. Individualized treatment decisions should be made in consultation with the patient, considering the severity of AA and available treatment options. Regular monitoring of blood counts and other relevant laboratory tests is often recommended during treatment with Osimertinib to detect any potential hematological abnormalities.

The occurrence of AA in non-metastatic cases would expand our understanding of the potential hematological side effects of Osimertinib beyond its typical association with metastatic disease. It suggests that factors other than metastasis, such as the drug itself or patient-specific factors, may contribute to the development of AA.

## Conclusion

Up to our knowledge, this case report documents the first recorded occurrence of AA associated with adjuvant Osimertinib therapy. The rarity of this adverse event highlights the importance of healthcare professionals diligently monitoring patients receiving Osimertinib for any signs of hematological toxicity, including pancytopenia. It is essential to promptly identify and appropriately manage these symptoms to achieve the best possible outcomes for patients. Further research is needed to explore risk factors, underlying mechanisms, and optimal management strategies for Osimertinib-induced AA in the adjuvant treatment context.

## Data availability statement

The original contributions presented in the study are included in the article/supplementary material. Further inquiries can be directed to the corresponding author.

## Ethics statement

Written consent was obtained from the patient for research and data publication. The case report was approved by the Hamad Medical Corporation’s Medical Research Center under the number: 04-23-388.

## Author contributions

AA: Data curation, Writing – original draft, Writing – review & editing. SK: Writing – original draft, Writing – review & editing. NO: Writing – original draft, Writing – review & editing. HA: Writing – review & editing. YE: Writing – review & editing. RM: Writing – review & editing.
